# Functional localization of the human color center by decreased water displacement using diffusion‐weighted fMRI

**DOI:** 10.1002/brb3.408

**Published:** 2015-10-14

**Authors:** Rebecca J. Williams, David C. Reutens, Julia Hocking

**Affiliations:** ^1^ Centre for Advanced ImagingThe University of QueenslandSt LuciaQld4067Australia; ^2^ Queensland Brain InstituteThe University of QueenslandSt LuciaQld4067Australia; ^3^ Centre for Clinical ResearchThe University of QueenslandBrisbaneQld4006Australia; ^4^ Hotchkiss Brain Institute and Department of RadiologyUniversity of CalgaryCalgaryAB T2N 4N1Canada; ^5^ School of Psychology and CounsellingQueensland University of TechnologyKelvin GroveQld4059Australia

**Keywords:** Area V4, BOLD, DfMRI, diffusion MRI, fMRI, magnetic resonance imaging

## Abstract

**Introduction:**

Decreased water displacement following increased neural activity has been observed using diffusion‐weighted functional MRI (DfMRI) at high *b*‐values. The physiological mechanisms underlying the diffusion signal change may be unique from the standard blood oxygenation level‐dependent (BOLD) contrast and closer to the source of neural activity. Whether DfMRI reflects neural activity more directly than BOLD outside the primary cerebral regions remains unclear.

**Methods:**

Colored and achromatic Mondrian visual stimuli were statistically contrasted to functionally localize the human color center Area V4 in neurologically intact adults. Spatial and temporal properties of DfMRI and BOLD activation were examined across regions of the visual cortex.

**Results:**

At the individual level, DfMRI activation patterns showed greater spatial specificity to V4 than BOLD. The BOLD activation patterns were more prominent in the primary visual cortex than DfMRI, where activation was localized to the ventral temporal lobe. Temporally, the diffusion signal change in V4 and V1 both preceded the corresponding hemodynamic response, however the early diffusion signal change was more evident in V1.

**Conclusions:**

DfMRI may be of use in imaging applications implementing cognitive subtraction paradigms, and where highly precise individual functional localization is required.

## Introduction

Sensitivity to neural activity‐induced changes in blood susceptibility has been the mainstay of functional magnetic resonance imaging (fMRI). Blood oxygenation level‐dependent (BOLD) signal changes are dependent on vascular and metabolic changes coupled to neural activity, although the mechanisms relating these processes remain unclear (Hillman 2014). This prevailing limitation reduces the accuracy with which the BOLD signal can be interpreted, as this requires a comprehensive understanding of how neural activity modulates cerebral blood flow and metabolism. In the instance of neurological disease, aging or modifications in baseline cerebral blood flow or metabolism, an altered BOLD response can ambiguously reflect alterations in either underlying neural activity or neurovascular regulation (Hamilton et al. 2010). Investigations into novel imaging techniques aim to improve sensitivity to physiological sources more specific to neuronal activity as a means to address these limitations associated with BOLD.

Diffusion‐weighted fMRI (DfMRI) has gained attention as one such technique reliant on sources distinct from blood oxygen changes (Aso et al. 2009; Le Bihan et al. 2006; Williams et al. 2014). Evidence demonstrating the continued presence of diffusion signal changes following the inhibition of neurovascular coupling has provided support for this distinct signal source (Tsurugizawa et al. 2013). This is in agreement with studies identifying restrictions in water diffusion induced by neuronal activity in *ex vivo* samples devoid of vasculature (Kohno et al. 2009; Tirosh and Nevo 2013). Despite this, whether DfMRI activation in vivo reflects neural activity more directly than BOLD remains unclear. Diffusion‐weighted imaging is inherently sensitive to BOLD signal changes, and the extent to which BOLD sources contribute to the overall DfMRI signal continues to be the subject of controversy (Miller et al. 2007; Autio et al. 2011; Kuroiwa et al. 2014). Establishing whether DfMRI is able to localize neural activity more directly than BOLD is critical to the validation and implementation of this novel technique for human brain mapping applications.

To characterize the temporal properties of DfMRI, Aso et al. (2013) extracted the diffusion signal change in V1, the parietal lobe and the inferior occipital cortex. These authors found that the diffusion signal temporally preceded the corresponding BOLD response in all three explored cortical regions. While the early diffusion response is indicative of unique physiological mechanisms to BOLD, little attention has been paid to the spatial properties of DfMRI activation. Characterizing the spatial properties of activation patterns across multiple brain regions is essential to determine whether DfMRI generally reflects physiological sources distinct from BOLD, and to establish the application of this technique in the context of cognitive paradigms. Functional imaging studies of cognition typically explore the effects of multiple low‐contrast experimental conditions that give rise to small signal changes. Because DfMRI suffers from lower signal‐to‐noise ratio (SNR) than BOLD, it is of interest to researchers in the cognitive neurosciences to characterize diffusion signal changes arising from the subtraction of minimally varying control and experimental conditions.

The aim of this study was to explore activation patterns obtained with DfMRI compared to BOLD outside of V1 using a cognitive subtraction task. We implemented a task designed to functionally localize the human color center Area V4. This region is ideal for assessing spatial specificity as previous research has verified its location along the ventral occipito‐temporal cortex and its strong correspondence with the lateral collateral sulcus, neighbored by the posterior fusiform and lingual gyri (McKeefry and Zeki 1997; Bartels and Zeki 2000). Because the functional localization of V4 requires detection of signal changes arising from the comparison of multiple low‐contrast experimental conditions, DfMRI in the context of cognitive tasks can be assessed. Evaluation of activation patterns was performed in terms of first‐ and second‐level activation maps, Euclidean distance between first‐level maxima, signal amplitudes, and temporal profiles.

## Methods and Materials

### Participants

Ten adults aged 19–40 years (4 female; mean age 26.2 years) gave written informed consent to participate in this study. They reported no history of neurological illness or injury, and had normal or corrected‐to‐normal vision. All participants passed an Ishihara test for color blindness prior to the commencement of the study (Hardy et al. 1945; Crognale et al. 2013). This study was approved by the University of Queensland Medical Research Ethics Committee for human studies.

### Stimulus design

The stimuli replicated previous positron emission tomography (PET) and BOLD fMRI imaging studies using the Mondrian paradigm to localize the human color center (Lueck et al. 1989; Zeki et al. 1991; McKeefry and Zeki 1997). The stimuli were created using the Psychophysics Toolbox (Brainard 1997; RRID:rid_000041) running on MATLAB (The Mathworks, Sherborne, MA, USA) and back‐projected onto an LCD screen located inside the bore of the scanner. The experimental condition consisted of six colored rectangles organized in an abstract ‘Mondrian’ pattern so that no recognizable object or scene could be detected. The colors included yellow, green, blue, red, cyan, and magenta. No single color was surrounded by any other single color, due to contextual effects in color processing (Shapley and Hawken 2011). An isoluminant achromatic control condition consisted of a Mondrian pattern identical to the colored, with the colors converted to grayscale to maintain luminance. Both chromatic and achromatic Mondrian conditions alternated with a blank ‘pattern offset’ at 1 Hz. The pattern offset consisted of a blank colored (chromatic condition) or grayscale (achromatic condition) screen equal in luminance to the Mondrian patterns.

### Experimental procedure

Prior to the commencement of scanning, participants completed a heterochromatic flicker photometry task to set isoluminance between colors and the baseline (Kaiser 1988). The following procedure was performed six times, once for each color in the chromatic Mondrian condition. A centrally located circle flickered at 15 Hz between color and a mean gray. The participant adjusted the luminance of the color until the minimum flicker was perceived. The subject‐specific brightness of the color at minimum flicker was recorded and used to set the luminance of the color for the chromatic Mondrian condition. This was converted to grayscale for the achromatic Mondrian condition. The mean gray was used for the baseline condition. Because the success of this task was dependent on environmental conditions such as lighting and visual angle, all participants completed this task under the same viewing conditions as the experimental procedure, that is in the scanner prior to the commencement of image acquisition.

**Figure 1:**
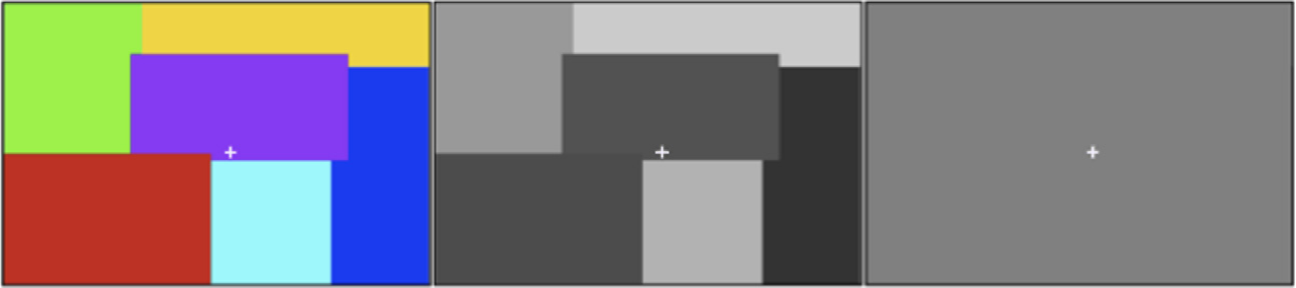
An example of the colored Mondrian (left), achromatic Mondrian (center), and blank baseline (right) screens viewed by the participants. All conditions were isoluminant as determined on an individual basis using the heterochromatic flicker photometry task (Kaiser 1988).

The block length of the two Mondrian experimental conditions was 8 sec. These experimental conditions were interspersed with a baseline condition consisting of a blank isoluminant gray screen. A schematic example of the stimuli used is shown in Figure 1. The duration of the baseline presentation was 24 sec. A white, centrally located fixation cross was consistently present in all three conditions. Participants made a right‐handed button press at the start and the end of every experimental block, with one button corresponding to the chromatic Mondrian condition and another indicating the achromatic Mondrian condition. These behavioral data were collected to ensure participant alertness. There were nine experimental blocks per run, with a total of 45 blocks each for the chromatic and achromatic conditions across 10 DfMRI runs, and nine blocks each for the 2 BOLD runs. The length of each run was 5 min.

### Data acquisition

All images were acquired on a Siemens 3 T TIM Trio (Siemens Medical Solutions, Erlangen, Germany) with a 12‐channel birdcage head coil. Head padding was inserted to minimize movement, and participants were carefully instructed to remain stationary. For every scan session, there were 10 runs of DfMRI and 2 of BOLD collected, with the duration of each run being 5 min. This ratio was performed to increase SNR of DfMRI, in keeping with previous work (Aso et al. 2009). The DfMRI acquisition was a twice‐refocused spin‐echo echo‐planar image (EPI) sequence, with diffusion sensitization attained by the addition of an interleaved pair of bipolar magnetic field gradients with a *b*‐value of 1800 mm/sec^2^ (Le Bihan et al. 2006). Images sensitized to BOLD contrast were acquired with a T_2_*‐weighted EPI sequence. The TR was 1500 msec for both functional sequence types, and the TE was 92 and 35 msec for DfMRI and BOLD, respectively. The voxel size was 3 × 3 × 3 mm with 10 slices separated by a 50% gap acquired in an interleaved order. In each functional run, 200 partial brain volumes were acquired. The functional volumes were aligned with the inferior temporal gyrus, ensuring total coverage of the temporal and occipital lobes. A high‐resolution T_1_ anatomical image was also collected for each scan session (TR = 1900 msec, TE = 2.32 msec, FOV = 230 × 230, 0.9 mm^3^ isotropic voxels). The total acquisition time for each scan session was 64 min.

### Data analysis

Images were analyzed using Statistical Parametric Mapping 8 (SPM8) (Wellcome Trust Centre for Neuroimaging, London UK; RRID:nif‐0000‐00343) running on MATLAB. Images were initially slice time corrected to the mid slice in the acquisition order, and realigned and resliced using a six‐parameter rigid body spatial transformation (Friston et al. 1995). The structural scan was coregistered to the mean functional image for each participant, and normalized to the MNI template using the Unified Segmentation algorithm (Ashburner and Friston 2005). Visual inspection ensured coregistration accuracy between DfMRI and anatomical images for all participants. Images were spatially smoothed using a 6 mm FWHM Gaussian kernel. All further quantitative measures were analyzed using IBM SPSS Statistics v20 (IBM Corp., Armonk, NY) and MATLAB. BOLD and DfMRI data were analyzed separately.

### First‐ and second‐level activation maps

First‐level statistical analysis of the BOLD data modeled the effects of the chromatic and the achromatic Mondrians independently by convolving the onset times with the canonical HRF. Six realignment parameters corresponding to translation and rotation were entered into the model as regressors of no interest. The statistical analysis of the DfMRI data was performed using the same procedure as the BOLD data; however, to increase the signal sensitivity for DfMRI, these data were modeled with the diffusion‐hemodynamic response function (DhRF) defined by Aso et al. (2009). Both within‐subjects first‐level and between‐subjects second‐level statistical analyses were performed. The contrasts of interest at the first‐level consisted of each Mondrian condition relative to the other Mondrian and baseline conditions (‘color > all’ and ‘achromatic > all’). These contrast images were then entered into random‐effects second‐level analyses performed separately for DfMRI and BOLD, including one‐way *t*‐tests identifying the group effects of each Mondrian condition alone, and paired *t*‐tests to compare color and achromatic Mondrian conditions. For all analyses and conditions, unless otherwise stated, whole‐volume searches were implemented with contrast images thresholded at *P *<* *0.05 familywise error (FWE) corrected for multiple comparisons for BOLD, and *P *<* *0.001 uncorrected for DfMRI.

### Euclidean distance between maxima

The aim of the Euclidean distance analysis was to examine the location of the colored Mondrian peak voxels obtained with DfMRI and BOLD to a defined anatomical region encompassing Area V4. To achieve this aim, analyses were performed on maxima extracted from individual statistical parametric maps (SPMs) for the contrast of ‘color > all’. The coordinates of activated voxels obtained from whole‐brain SPMs were extracted and compared to the coordinates of activated voxels obtained from SPMs which were inclusively masked. This mask covered V4 bilaterally and was developed from cytoarchitectonic maps of Brodmann's areas using the SPM Anatomy Toolbox (Eickhoff et al. 2005; RRID:nif‐0000‐10477).

There were four experimental conditions in the Euclidean distance analysis: whole‐brain for DfMRI (DfMRI_whole_) and BOLD (BOLD_whole_), and V4 inclusively masked DfMRI (DfMRI_V4_) and BOLD (BOLD_V4_). The maxima included in the Euclidean distance analyses were those reported by the default SPM8 setting, where the three peak maxima separated by a minimum distance of 8 mm within each cluster are reported. For both BOLD (BOLD_V4_ and BOLD_whole_) statistical maps, the threshold was set to *P *<* *0.05 FWE corrected for multiple comparisons. The threshold was reduced to *P *<* *0.001 uncorrected for every DfMRI SPM. To determine if there was a difference between the four conditions in terms of the number of maxima included in the analysis, a one‐way repeated measures analysis of variance (ANOVA) was performed on the number of maxima, with post hoc comparisons using Fisher's least significant difference.

The distances between homologous peak maxima were calculated between paired comparisons of the four experimental conditions. Paired comparisons were performed within‐sequence (DfMRI_V4_–DfMRI_whole_; BOLD_V4_–BOLD_whole_), with the sequence demonstrating the shorter mean Euclidean distance indicating activation more spatially specific to V4. Paired comparisons between‐sequence (DfMRI_V4_–BOLD_V4_; DfMRI_whole_–BOLD_whole_) were also made to determine overlap between DfMRI and BOLD. Homologous maxima were defined as those with the shortest distance between them. Pairs of peaks separated by a distance greater than one standard deviation from the mean of each comparison were excluded from the analysis as outliers. For each participant, the mean distance between homologous maxima for each of the four conditions were entered into paired *t*‐tests comparing each comparison to all others. All distances between homologous maxima for each of the four comparisons also underwent a frequency analysis. The purpose of the frequency analysis was to examine the most commonly occurring distance between maxima in each comparison. This was to determine how the number of homologous maxima in each condition influenced the mean distance in paired comparisons.

### Signal amplitude

To determine the differences in response amplitude between DfMRI and BOLD to color and achromatic Mondrian stimuli, the percent signal change within V1 was calculated and quantitatively compared. V1 was chosen to represent signal change as it was expected to activate equivalently to the colored and achromatic Mondrians. While cells with a preference for color have been identified within V1 (Johnson et al. 2001), the methodological approach used here ensured that the voxels selected for calculating percent signal change were optimized for each experimental condition independently. Each of the four contrasts of interest (‘color > all’ for DfMRI and BOLD; ‘achromatic > all’ for DfMRI and BOLD) was entered into separate one‐sample *t*‐tests at the group level. The resultant statistical maps were inclusively masked with an anatomical mask corresponding to Brodmann's Area 17, defined by cytoarchitectonic maps (Eickhoff et al. 2005). The coordinates of the voxel with the highest *t*‐value at the group level represented the center of a 10 mm sphere small volume search within each participant's corresponding first‐level contrast image. The peak voxel within the sphere was extracted and its percent signal change calculated using the MarsBaR Region of Interest toolbox for SPM (Brett et al. 2002; RRID:nlx_155806). In cases where no suprathreshold voxels were identified within the 10 mm sphere, the closest individual peak to group peak was used. Bivariate correlation analyses were performed to assess for relationships between conditions.

### Temporal response profiles

The time‐courses were analyzed to determine if a consistent temporal precedence for DfMRI relative to BOLD is found in both V1 and V4. To achieve this, the time‐to‐peaks (TTP) of the response in V4 and in V1 were quantitatively compared between DfMRI and BOLD.

To extract the diffusion and the BOLD response in areas V4 and V1, first‐level analyses were performed specifically for the temporal profile analysis, which modeled both DfMRI and BOLD data using the canonical hemodynamic response function and its time and dispersion derivatives. Including the derivatives in the model accounted for temporal and dispersion variations in the response (Friston et al. 1998), while keeping a consistent model between datasets.

To consider the temporal profile of diffusion and BOLD responses in areas V4 and V1, the contrast images corresponding to ‘color > all’ from the analyses modeled with the canonical HRF its derivatives were entered into separate second‐level one‐sample *t*‐tests for DfMRI and BOLD. The group statistical image was inclusively masked with the V4 and V1 masks separately, to obtain group peaks within these two visual areas. For each participant, the time‐courses of the peak voxels closest to the group V1 and V4 peaks were extracted for each of the four conditions (DfMRI_V4_, DfMRI_V1_, BOLD_V4_, and BOLD_V1_). This was achieved by separately using the V1 and V4 group peak voxels as the center of a 10 mm sphere volume of interest (VOI) in the individual SPMs of the corresponding ‘color > all’ contrast. The time‐series of all activated voxels within the sphere were extracted for all runs of data and used in the present profile analysis. The TTP was determined directly from these data. The extracted time‐courses were initially interpolated from scans to milliseconds. The times corresponding to the onset of each colored Mondrian stimulation period and the subsequent baseline period (32 sec of data in total) were extracted from each VOI, scaled (between 0 and 1) and averaged across runs to provide a robust subject‐specific response. The TTP was calculated from each subject‐specific response and defined as the time point when the signal intensity reached its maximum within the 32‐sec period. For each subject, the TTPs for each of the four conditions were entered into statistical comparison tests. Because the distribution of TTPs did not conform to normality, paired comparisons were performed using a Wilcoxon signed‐rank test. Within‐sequence (DfMRI_V1_ vs. DfMRI_V4_; BOLD_V1_ vs. BOLD_V4_) and between‐sequence (DfMRI_V1_ vs. BOLD_V1_; DfMRI_V4_ vs. BOLD_V4_) paired comparisons were performed.

## Results

### First‐ and second‐level activation maps

**Figure 2:**
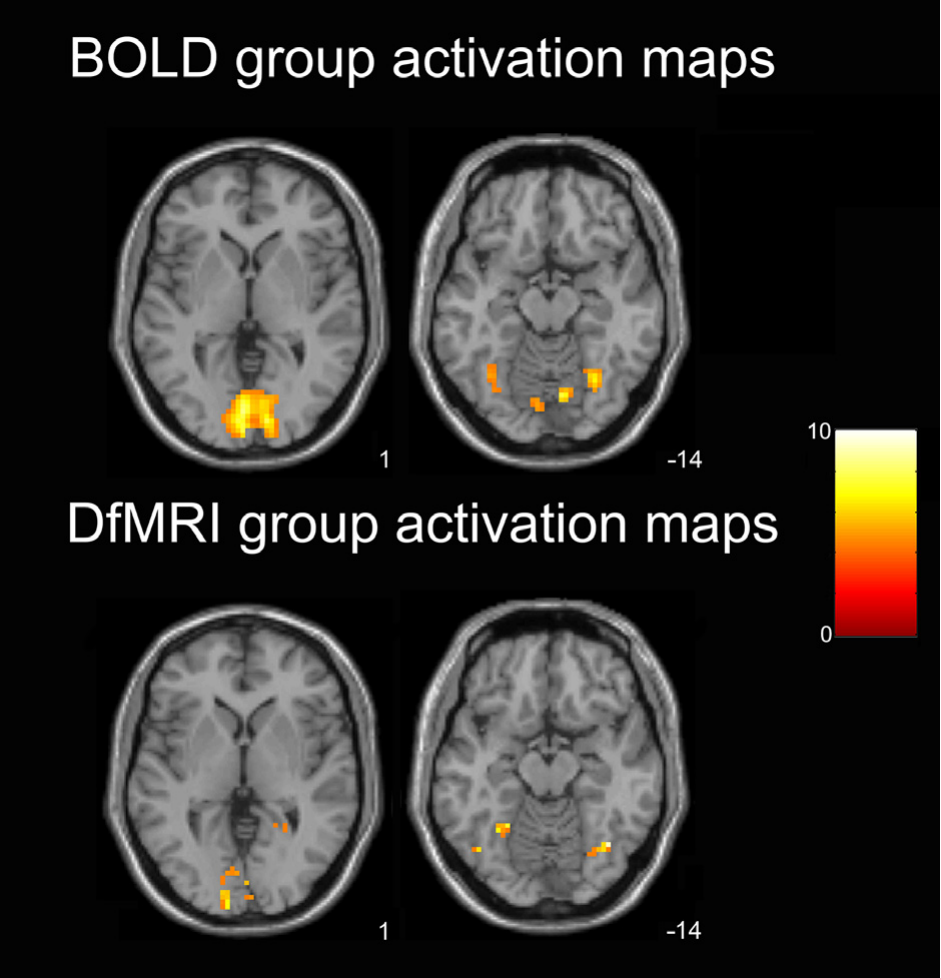
One‐sample *t*‐test activation maps showing group activation for the color Mondrian condition. Axial slices shown at peak voxel level for BOLD (z = 1) and DfMRI (z = −14). SPMs thresholded at *P *<* *0.001 uncorrected for multiple comparisons and overlaid into MNI T_1_‐weighted template image. Color bar indicates *t*‐values.

Individual statistical maps showing the effects of ‘color > all’ revealed consistent brain activation in the ventral visual‐processing stream for both DfMRI and BOLD, however, lower signal detection for DfMRI was evident through smaller cluster sizes and lower statistical values. At the second‐level, one‐sample *t*‐tests identifying the group effects of the color Mondrian condition showed higher statistical values for DfMRI than BOLD at the peak voxel. The peak voxel for DfMRI was located within the right fusiform gyrus [39, −64, −14], *z* = 4.45, *P* = 0.04 FWE corrected. Conversely, the BOLD peak voxel in the color > all one‐sample *t*‐test was located within the primary visual cortex and was not significant at a corrected level, [−3, −82, 1], *z* = 4.31, *P* = 0.07 FWE corrected. Figure 2 demonstrates the one‐sample *t*‐test group activation maps in the axial plane. Both BOLD and DfMRI activation maps are shown at the level of the peak voxel for each sequence (z = 1 and −14 for BOLD and DfMRI, respectively). This figure demonstrates that compared to BOLD, DfMRI has smaller cluster sizes and less activation in V1 (z = 1). But within the inferior temporal lobe (z = −14) DfMRI shows comparable cluster sizes to BOLD and higher statistical scores in the inferior temporal/fusiform region corresponding to V4.

**Figure 3:**
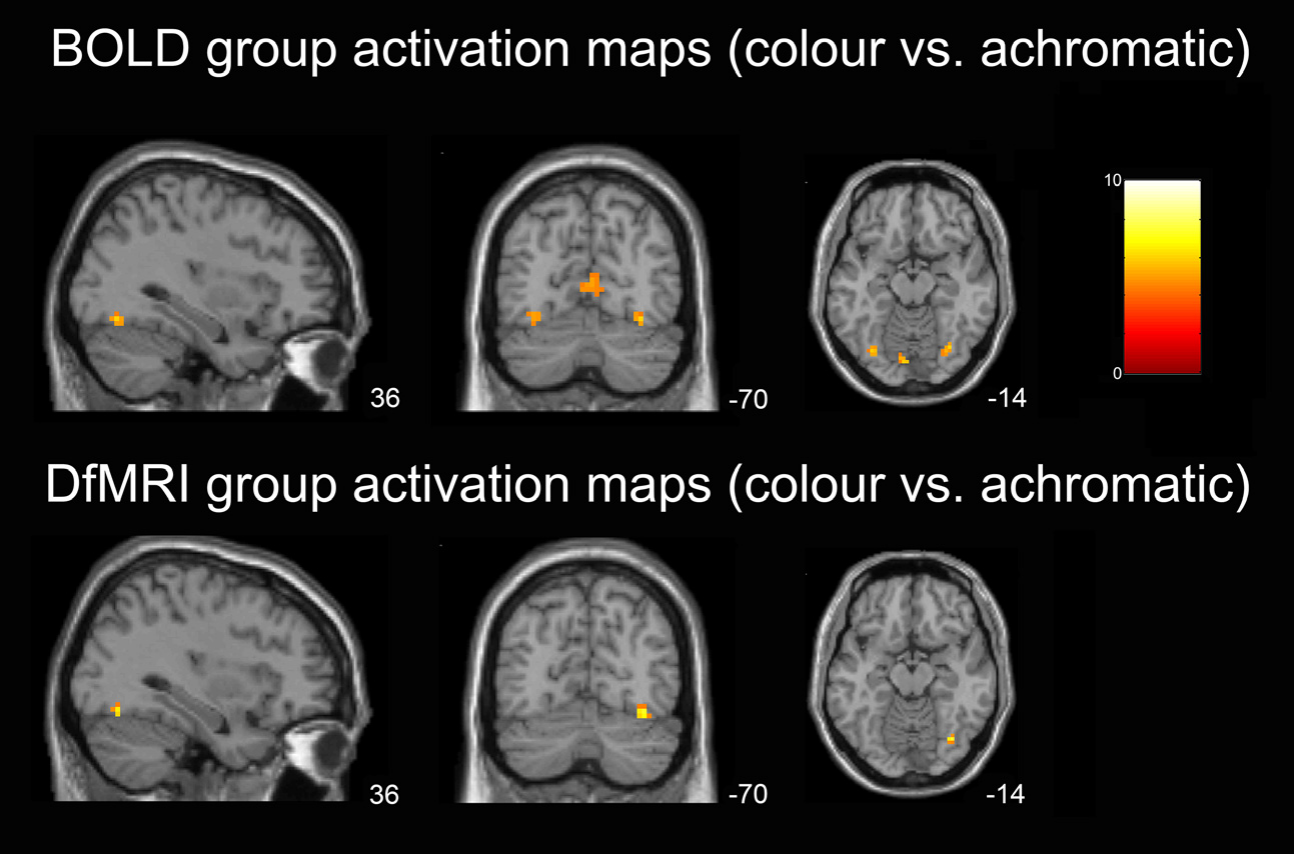
Paired‐samples *t*‐test activation maps showing group activation for color versus achromatic Mondrian conditions. BOLD demonstrated larger cluster sizes in V1, and more suprathreshold bilateral V4 activation than DfMRI. There was V1 activation for DfMRI also, although this is not evident here due to the level of the slices shown. Slices shown at the level of peak V4 activation for DfMRI, which was within the right hemisphere. SPMs overlaid onto MNI T_1_‐weighted template image and thresholded at *P *<* *0.001 uncorrected. MNI coordinates shown. Color bar indicates *t*‐values.

The paired *t*‐tests showed significant group effects for color > achromatic Mondrians for both BOLD and DfMRI. Activation peaks were found in the primary visual and lateral occipito‐temporal cortices for both datasets. For BOLD, the peak cluster encompassed the posterior occipital lobe, with the peak voxel located within the primary visual cortex [−9, 88, −2], *z* = 5.5, *P *<* *0.0005 FWE corrected. The second peak cluster, located in the ventral occipito‐temporal cortex, showed a peak voxel located within V4 [33, −70, −14], *z* = 3.7, *P *<* *0.0005 uncorrected (nonsignificant at FWE corrected *P*). For DfMRI a similar pattern was found, with the most highly significant cluster found within the posterior occipital lobe. The peak voxel for this cluster was located ventral to the primary visual cortex [9, −82, −2], *z* = 4.2, *P *<* *0.0005 uncorrected. Similar to BOLD, the second peak cluster was located within the ventral occipito‐temporal cortex for DfMRI, with the peak maxima located in V4 [36, −70, −14]. This voxel reached a slightly higher statistical value for the DfMRI analysis than the BOLD results, although it too failed to reach significance at a corrected level, *z* = 4.04, *P *<* *0.0005 uncorrected. The paired *t*‐test activation results for DfMRI and BOLD are shown in Figure 3. As demonstrated in this figure, shown at level of the peak DfMRI voxel in V4, cluster sizes were overall larger for BOLD in both V1 and V4. Significant voxels within the right V4 showed high statistical values for DfMRI.

### Euclidean distance between maxima

The one‐way ANOVA performed on the total number of maxima showed that there was a significant difference between conditions, *F*[1,8] = 9.4, *P *=* *0.02. Paired comparisons revealed that the DfMRI_v4_ condition (*M* = 2.8, ± 1.9) had significantly fewer peaks than BOLD_whole_ (*M* = 6.7, ± 2.5, *P *=* *0.01) and BOLD_v4_ (*M* = 5.9, ± 2.6, *P *=* *0.007). There was no difference between DfMRI_whole_ (*M* = 4.9, ± 4.1) and all other conditions. The number of outliers removed from the DfMRI_whole_–BOLD_whole_ comparison was 4 (17.4%); 4 (26.6%) for the DfMRI_V4_–DfMRI_whole_ comparison; 3 (13%) for the DfMRI_V4_–BOLD_V4_ comparison and 4 (18.2%) for BOLD_V4_–BOLD_whole_.

The conditions with the shortest Euclidean distance between maxima were DfMRI_V4_–DfMRI_whole_, followed by BOLD_V4_–BOLD_whole_. The comparison with the greatest distance between maxima was found for DfMRI_whole_–BOLD_whole_ followed by DfMRI_V4_–BOLD_V4._ Paired samples *t*‐tests showed that the distance between the two DfMRI conditions (DfMRI_V4_–DfMRI_whole_) was significantly shorter than all other paired comparisons (all *P *<* *0.004). All distances are shown in Table 1. For the frequency analysis performed on all distances between homologous maxima, the DfMRI_V4_–DfMRI_whole_ comparison most commonly reported 0 mm between maxima (58.3%, range = 0–4.2 mm), as did the BOLD_V4_–BOLD_whole_ comparison (33.3%, range = 0–19.2 mm). The most frequently occurring distances between DfMRI_V4_ and BOLD_V4_ homologous maxima were 6.71 and 7.35 mm (20% each, range = 0–9.9 mm). The most frequent distance for the DfMRI_whole_ and BOLD_whole_ comparison was 7.35 mm (15.8%, range = 3–16.2 mm).

**Table 1:** Euclidean distance (mm) between maxima for DfMRI and BOLD for the whole‐volume and for within V4 only

Pt	DfMRI_whole_ BOLD_whole_	DfMRI_V4_ BOLD_V4_	DfMRI_V4_ DfMRI_whole_	BOLD_V4_ BOLD_whole_
1	7.3	6.7	0	6.5
2	NA	8.3	3.0	7.9
3	9.5	4.2	3.0	4.3
4	16.2	6.6	NA	0
5	8.5	6.3	2.1	9.6
6	6.0	4.4	1.0	8.6
7	9.0	6.7	1.5	2.1
8	6	5.8	0	4.9
9	15.0	5.2	0	NA
Mean	9.7 (± 3.9)	6.0 (± 1.3)	1.3 (± 1.3)	5.5 (± 3.3)

NA, no homologous maxima available; Pt, participant.

### Signal amplitude

One participant was removed from the analysis for failing to show significant activation in V1. The greatest percent signal change was attained for the BOLD color condition (*M* = 2.1 ± 0.76%) followed by the BOLD achromatic condition (*M* = 0.93 ± 0.51%). The two BOLD conditions also showed the greatest range of percentages, from 1.7 to 3.86% for BOLD color, and 0.43 to 1.85% for BOLD achromatic. The achromatic DfMRI condition was slightly higher (*M* = 0.85 ± 0.13%) than the DfMRI color condition (*M* = 0.82% ± 0.27%). The range of percentages was more restricted for DfMRI color (0.56–1.36%) and DfMRI achromatic (0.61–1%). The bivariate correlation analyses were all nonsignificant.

### Temporal response profiles

**Figure 4:**
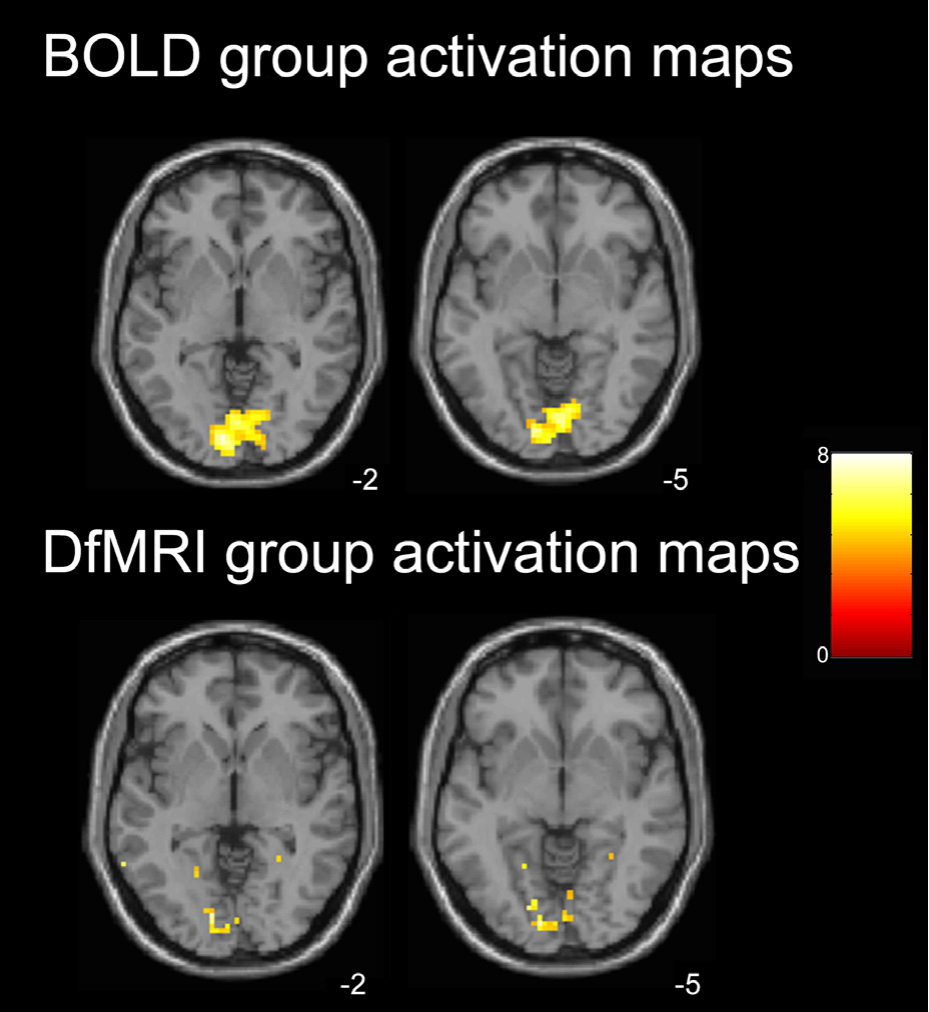
Group activation maps when both DfMRI and BOLD modeled with a common basis set (canonical HRF with temporal and dispersion derivatives). One‐sample *t*‐tests, color > all contrast. Axial slices shown at peak voxel level for BOLD (*z* = −2) and DfMRI (*z* = −5). SPMs thresholded at *P *<* *0.001 uncorrected for multiple comparisons and overlaid into MNI T_1_‐weighted template image. Color bar indicates *t*‐values.

The group‐level analyses resulting from the implementation of the canonical HRF with temporal and dispersion derivatives for both DfMRI and BOLD showed, for DfMRI, less activation than the previous group analyses using the DhRF template. As demonstrated in Figure 4, the contrast ‘color > all’ group activation maps using the canonical HRF derivative basis sets resulted in less group activation in the inferior temporal cortex for DfMRI, compared to the corresponding activation maps using the DhRF shown in Figure 2. The DfMRI peak voxel when using the canonical HRF basis set ([−12, −88, −5], *z *=* *4.1, *P *=* *0.17) was located posteriorly to the corresponding peak voxel obtained using the DhRF model, with these results displayed in the ‘First and second‐level activation maps’ section above. For BOLD, accounting for the temporal and dispersion derivatives in the model resulted in only minor changes in activation patterns. The whole‐brain peak voxel for BOLD (‘color > all’ contrast) when modeling the HRF derivatives ([−9, −88, −2], *z *=* *4.1, *P *=* *0.12), similar to the corresponding peak in the above, remained in the posterior portion of the occipital lobe.

For the extraction of the time‐courses, these activation maps were inclusively masked with anatomical ROIs. The group peak voxel within V4 for DfMRI using the HRF template was [−21, −67, −14], and [30, −67, −14] for BOLD. For V1, the group peak voxel was [−9, −88, 7] for DfMRI and [−9, −88, −2] for BOLD. One participant was removed from the temporal profiles analysis as an outlier, due to a TTP greater than two standard deviations from the mean. The mean DfMRI TTP within V1 (M = 6.1 ± 1.6 sec) and V4 (7.6 ± 2.6 sec) preceded the BOLD response for both cortical regions (M = 9.3 ± 2.6 and M = 8.5 ± 2.0 sec for V1 and V4, respectively). The paired comparisons performed on the individual TTPs showed a significant precedence for DfMRI relative to BOLD in V1, *z *=* *2.0, *P *=* *0.05. No other paired comparison reached significance. Many participants showed a TTP that was shorter than the length of the 8‐sec stimulation period, particularly for DfMRI time courses extracted from V1. The most restricted range of TTPs across participants was found for DfMRI_V1_. For this condition, the minimum was 4.0 sec and the maximum TTP was 8.6 sec. For DfMRI_V4_, the TTPs showed a greater range across participants (4.0–11.0 sec). The TTPs ranged between 4.8–13.1 sec for BOLD_V1_ and 5.6–10.5 sec for BOLD_V4_. Table 2 shows the TTP for each participant.

**Table 2:** Time‐to‐peak of the temporal profile with V1 and V4 for DfMRI and BOLD

Participant	DfMRI_V1_	DfMRI_V4_	BOLD_V1_	BOLD_V4_
1	8.0	8.5	9.9	9.7
2	8.6	6.3	10.1	10.5
3	5.6	4.8	11.8	7.8
4	4.1	10.3	9.3	7.6
5	5.5	4.0	10.0	10.3
6	5.4	8.5	8.6	8.6
7	4.0	5.1	4.8	5.6
8	7.1	11.0	13.1	5.6
9	6.6	10.2	6.0	10.5
Mean	6.1 (± 1.6)	7.6 (± 2.6)	9.3 (± 2.6)	8.5 (± 2.0)

## Discussion

In this study, the spatiotemporal properties of DfMRI were investigated using a Mondrian paradigm designed to functionally localize the human color center V4. We demonstrated that DfMRI activation can be detected within the anticipated region of the fusiform gyrus, and in individual analyses, appears to be more spatially localized to this region compared to concordant BOLD activation, which was more dispersed throughout the visual cortices. This is the first study to examine the spatial specificity of DfMRI activation to neural activity outside of V1. Localizing neural activity in V4 was achieved by contrasting multiple experimental conditions, indicating that DfMRI may be used in studies examining cognitive brain activity.

### DfMRI and studies of cognition

Diffusion‐weighted fMRI showed less sensitivity to signal change than BOLD, with the DfMRI individual activation maps showing the least activation when the effects of the colored Mondrian condition was contrasted to all other conditions. Despite this, the DfMRI activation was consistently located within the expected locus of neural activity. This finding supports the efficacy of DfMRI to functionally localize discrete regions of cortex outside the primary sensory regions using a more sensitive cognitive subtraction design. Currently, in vivo human DfMRI studies have mainly implemented simple visual stimulations contrasting a checkerboard stimulus with a blank baseline (Darquie et al. 2001; Aso et al. 2009; Le Bihan et al. 2006; Miller et al. 2007; Kohno et al. 2009; Williams et al. 2014). Aso et al. (2013) were the first to observe a diffusion response outside the primary sensory domain. These authors performed a working back memory task using DfMRI, contrasting the 2‐back paradigm with a rest baseline. These authors reported both diffusion and BOLD activation in the parietal lobe to this task. The findings of this study are in line with this previous work, demonstrating that DfMRI may be used to detect signal changes arising from low‐contrast experimental conditions. This study provides further support for the use of DfMRI in cognitive experiments by showing that the diffusion response is spatially localized to the activated region of cortex.

It has previously been shown using BOLD fMRI that a colored Mondrian could be ‘added’ to an achromatic Mondrian, and subtracting the achromatic from the color Mondrian condition would localize the cortical color center (McKeefry and Zeki 1997; Bartels and Zeki 2000; Harada et al. 2009). Here, it was shown that DfMRI also reveals the color center using this cognitive subtraction paradigm. Moreover, the DfMRI color center was shown to be spatially distinct from the peak BOLD activation, and was more consistent between individuals as demonstrated by the increased statistical values within V4 at the second‐level. These group‐level statistical scores, however, were only slightly higher for DfMRI and both sequence types failed to reach significance at a corrected level within V4 at the group level. This may reflect slight individual variations in the location of V4, which has shown minor spatial differences between individuals particularly along the anterior‐posterior axis of the fusiform region (McKeefry and Zeki 1997).

### Signal magnitude changes differed between BOLD and DfMRI

Colored and achromatic Mondrian stimuli resulted in differing magnitude changes within V1 for BOLD, with the colored Mondrians invoking the larger signal change. This finding may be indicative of the neural physiology underlying BOLD. Because of its sensitivity to deoxyhemoglobin concentration changes (Ogawa et al. 1990), which are dependent on neural activity‐induced changes in cerebral blood flow, cerebral blood volume and cerebral metabolic rate of oxygen (Buxton et al. 2004; Mark et al. 2015), the BOLD signal magnitude is positively related to neural activity. Studies using electrophysiology have provided evidence for the association between the BOLD amplitude and the components of neural activity that it best represents. Recent studies observing the correlation between the BOLD signal and local field potentials (LFPs), which reflect mass extracellular activity in a region of cortex (Logothetis 2008), indicate a strong association between the BOLD magnitude and the gamma frequency band of LFPs (Kayser et al. 2004; Scholvinck et al. 2010; Magri et al. 2012). The high‐frequency gamma range has been associated with excitatory and inhibitory synaptic activity (Brunel and Wang 2003) and is believed to represent synchronous activity from large neural networks (Jensen et al. 2007). BOLD sensitivity to large neural networks may be one possible explanation for our finding that the colored Mondrians invoked a larger V1 BOLD signal magnitude than their achromatic counterparts. V4 was expected to activate preferably to the colored Mondrians, however, its connections to visual areas V3, V2, and V1 are extensive, including both feedback and feedforward communication (Ungerleider et al. 2008). Feedback communication from extrastriate regions does not affect neuronal spiking activity in the primary visual cortex, where firing activity is dependent on stimulation of the receptive field of the neuron (Budd 1998; Kim and Freeman 2014). Our finding of V1 BOLD activation, which was consistent with reported findings in previous color‐processing literature (McKeefry and Zeki 1997), may reflect feedback mechanisms associated with the wider ventral‐processing stream engaged for color processing, rather direct neural activity.

Unlike BOLD, the DfMRI signal magnitude in V1 was consistent for colored and achromatic Mondrians. One speculative explanation for these divergent findings is that the neurophysiology underlying DfMRI contrast, unlike BOLD, it not sensitive to feedback communication. Consistent with this argument are the previous findings by Tsurugizawa et al. (2013). In this animal study, DfMRI signal changes concordant with the induced neural activity were found under conditions of administered nitroprusside, which is known to induce neurovascular decoupling. The BOLD response, conversely, was mostly eliminated. While the present results cannot provide direct evidence for the neurophysiology responsible for the DfMRI response, they may suggest that the DfMRI signal is not dominated by BOLD contributions, yet rather reflect distinct physiology. The implication of this is the intriguing possibility that DfMRI may provide a more intimate view of neural activity within the framework of a larger network. Indeed, the highly localized V4 activation for DfMRI may indicate its sensitivity to this important node in the color‐processing stream, complementing the BOLD signal engaging the larger visual network. If confirmed, then the combination of DfMRI and BOLD may provide a powerful tool for research and clinical applications.

### Spatiotemporal differences between BOLD and DfMRI

The shortest distance between homologous maxima was observed between the masked and whole‐volume DfMRI conditions. This indicated that DfMRI activation was more localized within V4. However, an important consideration in the interpretation of these findings is the lower SNR and hence lower number of maxima attained for DfMRI. The frequency analysis showed that 0 mm was the most commonly occurring distance observed for both DfMRI_V4_/DfMRI_whole_ and BOLD_V4_/BOLD_whole_. The difference between these two comparisons was the range of distances attained, indicating that BOLD was similarly activating within V4 as well as outside the color region. These findings may be interpreted in relation to the statistical threshold used. In other words, would BOLD reveal a response comparable to DfMRI if the statistical threshold was set at a more conservative probability value? While it is important to consider this possibility, we believe that there is good evidence that changing the threshold would not result in equivalence between DfMRI and BOLD. The second‐level activation maps for DfMRI showed a slightly higher statistical value within V4 relative to BOLD, indicating that this region was more commonly activated across individuals for DfMRI, despite its lower sensitivity. The distance between DfMRI and BOLD maxima within V4 was the second highest, suggesting that the peak locations within V4 differed between the sequences. It therefore cannot be asserted that the differences between DfMRI and BOLD are solely reliant on SNR and thresholding. Rather, the present analysis supports the results of the analyses discussed above, which suggest that BOLD reflects the wider visual‐processing network whereas DfMRI reflects a more direct view of the locus of neural activity. However, it is important to note that further research investigating the reliability of the spatial activation patterns obtained for DfMRI is warranted.

When we compared the spatial properties of BOLD and DfMRI activation patterns in the first and second‐level analyses (Figs. 2 and 3) and the Euclidean distance analysis, we implemented convolution models optimized for each sequence type. That is, the canonical HRF for BOLD and the DhRF for DfMRI. The use of a common, canonical HRF basis set for modeling DfMRI and BOLD in the temporal profile analysis demonstrated some activation for DfMRI, as shown in Figure 4. Activation detected using this basis set for DfMRI peaked in the posterior occipital lobe, similar to BOLD. These findings lend support to some DfMRI sensitivity to BOLD effects. However, unlike the activation detected using the DhRF model, the peak voxel failed to reach significance at a corrected threshold when the canonical HRF basis set was used, showing an advantage of the DhRF model for DfMRI.

The temporal profiles in V1 and V4 for DfMRI were found to precede the BOLD response, yet only the V1 response was significantly shorter. Prior literature finding temporal equivalence between BOLD and DfMRI have cited this as evidence for dominance of the BOLD component in the diffusion signal (Goerke and Moller 2007; Yacoub et al. 2008; Rudrapatna et al. 2012). This nonsignificant difference between DfMRI and BOLD in the V4 time‐to‐peak may therefore indicate that the diffusion response in this brain region has a higher BOLD contribution relative to V1. However, a decreased sensitivity to diffusion signal change due to the lower SNR outside of V1 may also influence these analyses. The temporal precedence for DfMRI in V4 may reach significance with increased power. Aso et al. (2013) reported a temporal precedence for DfMRI in the parietal lobe. With 21 participants, Aso and colleagues had more power in their experimental design. These authors employed further temporal smoothing to the raw time courses using a moving‐average filter. This smoothing filter may increase SNR, however, it was not employed in the present analysis as it may also remove vital information. Evidently, the low SNR is a major limitation in DfMRI that may increase the hemodynamic component of the signal.

### Limitations and future directions

The results reported here reflect the comparison between a spin‐echo DfMRI sequence and a gradient‐echo BOLD sequence. A spin‐echo BOLD sequence may reduce signal variability associated with larger, distant draining veins and improve spatial localization to the capillaries (Hulvershorn et al. 2005), and should therefore be considered for future BOLD‐DfMRI comparisons. In this study, it was assumed that the experimental paradigm aiming to isolate the differences between colored and achromatic Mondrian conditions would result in the exclusion of BOLD activation attributed to draining veins, as this nonspecific activation would be common to all stimulus conditions. However, the use of spin‐echo BOLD should be carefully considered in future studies comparing the spatial specificity of BOLD and DfMRI, particularly where simple stimuli and experimental contrasts are employed. Another consideration is the image preprocessing implemented in this study. Future studies aiming to obtain highly precise spatial localization of BOLD and DfMRI activation should consider the necessity of spatial normalization and smoothing. These preprocessing steps were implemented here to obtain group activation maps, and were applied equivalently to both DfMRI and BOLD. Despite this, the effect of these processes on spatial localization must be considered. An important caveat for all future studies utilizing DfMRI is the low SNR, which may benefit from future research investigating the influence of field strength on the signal. Further studies should also be aware of system or vendor‐specific factors when implementing DfMRI, such as table vibrations due to diffusion gradients. Vibration intensity may differ across systems and vendors, and adequate testing of this is recommended prior to the commencement of a DfMRI study.

The value of DfMRI lies in its potentially unique physiological underpinnings. Further research verifying that its biological source is mainly distinct from BOLD is essential. However, if confirmed, DfMRI may overcome the limitations associated with identifying neural activity through an indirect hemodynamic surrogate. DfMRI may therefore provide an alternative to BOLD when highly precise mapping of neural activity is required, and where BOLD may fail due to its reliance on neurovascular coupling (Mark et al. 2015). Mapping neural activity in patients with cerebrovascular disease with fMRI is highly beneficial, as it offers insight into treatment progression and neural reorganization following injury. However, disrupted vasculature may ambiguously disrupt the BOLD signal. For instance, in ischemia, pathology resulting from vessel blockage may affect capillary blood flow regulation (Hall et al. 2014; MacDonald and Frayne 2015), reducing the ability with which a BOLD response can be accurately interpreted. In such situations, DfMRI and its dependence on a biological source distinct from BOLD may provide some benefit.

## Conclusions

In summary, the results of this study suggest that the diffusion response is spatially distinct from BOLD. It was shown here that the superior DfMRI spatial localization obtained within the primary visual cortex in previous research (Williams et al. 2014) can be extrapolated to the extrastriate cortex. The SNR, however, does decrease outside V1, which may influence analyses of the DfMRI response profile. The decreased SNR of DfMRI represents a major limitation in the continued application of this novel technique. DfMRI activation in V4 demonstrated somewhat higher interindividual consistency in spatial activation patterns and signal magnitude change, and a slight temporal precedence compared to BOLD. The pertinent conclusion of this study is that DfMRI appears to measure different neurophysiological components to BOLD, although further research determining the mechanisms representing the diffusion response is warranted.

## Conflict of Interest

None declared.
